# Authors’ response: Seasonality and effects of climatic exposures on community-acquired Legionnaires’ disease incidence, Italy, 2005 to 2023

**DOI:** 10.2807/1560-7917.ES.2026.31.29.2600606

**Published:** 2026-07-23

**Authors:** Antonio Sciurti, Maria Luisa Ricci, Maria Scaturro, Jessica Iera, Fabiola Mancini, Benedetta Bellini, Patrizio Pezzotti, on behalf of the authors of the original article

**Affiliations:** 1 Department of Public Health and Infectious Diseases, Sapienza University of Rome, Rome, Italy; 2 Department of Life Sciences, Health, and Health Professions, Link Campus University, Rome, Italy; 3 Department of Infectious Diseases, Istituto Superiore di Sanità, Rome, Italy; 4 ESCMID Study Group for *Legionella* Infections (ESGLI), Basel, Switzerland; 5 https://www.eurosurveillance.org/content/10.2807/1560-7917.ES.2026.31.12.2500712

**Keywords:** Legionella, Legionnaires’ disease, climate, temperature, rainfall, humidity, engineered water systems

**To the editor:** We thank the authors of the letter for their interest in our study about seasonality and climatic exposures effects on community-acquired Legionnaires’ disease (LD) in Italy, and for expanding on the potential role of the ecology of engineered water systems as a bridge between weather conditions and infection occurrence.

Our study shows that temperature and other climatic conditions are relevant drivers of community-acquired LD occurrence. The authors of the letter suggest that such effects are largely indirect, mediated by the ecology of engineered water systems. In our view, the distinction between direct or indirect effects is not central to the question we address, that is whether climatic exposures affect the risk of community-acquired LD. As with many climate-sensitive infectious diseases, they should be considered within a broader framework of complex interactions between susceptible human hosts and environmental conditions [1], which in this case include, but are not limited to, engineered water systems [2,3].

Even if engineered water systems constitute a relevant ecological interface for community-acquired LD, cases may derive from heterogeneous sources, including natural environments such as soil and ponds, from which viable *L. pneumophila* has been recovered [2,3]. Indeed, measured precipitation effects are consistent with direct aerosol dispersal from such varied sources [2,3]. Furthermore, a recent study investigating building water systems in four European cities found that both positive and negative associations between *Legionella* spp. and other microorganisms were consistently measured across all four cities, as well as across different buildings and water compartments [4]. These findings suggest the existence of ecological relationships that may be generalisable across different engineered water systems.

Nevertheless, we agree that climatic conditions alone capture only part of the phenomenon, and that integrating them with indicators of water system conditions and vulnerability could improve modelling of community-acquired LD occurrence and thereby better inform preventive strategies [5]. However, integrating population-scale LD surveillance with environmental microbiology and ecological monitoring of engineered water systems remains a challenge that should be addressed before such overarching modelling strategies can be developed. A more tractable, yet still ambitious, approach may be to quantify how single factors, including indicators of engineered systems conditions and natural reservoirs, affect LD risk and modify climatic effects.

Although our analysis addresses only part of the multifactorial mechanisms determining community-acquired LD risk, our results indicate that climatic conditions have been important drivers of the sustained increase in LD incidence observed in Italy over the last 20 years, with relevant implications for public health surveillance and prevention strategies.
